# Spectroscopic Characterization and Biological Activity of Hesperetin Schiff Bases and Their Cu(II) Complexes

**DOI:** 10.3390/ijms24010761

**Published:** 2023-01-01

**Authors:** Anna Sykuła, Adriana Nowak, Eugenio Garribba, Aliaksandr Dzeikala, Magdalena Rowińska-Żyrek, Justyna Czerwińska, Waldemar Maniukiewicz, Elżbieta Łodyga-Chruścińska

**Affiliations:** 1 Faculty of Biotechnology and Food Sciences, Institute of Natural Products and Cosmetics, Lodz University of Technology, Stefanowskiego 2/22, 90-537 Lodz, Poland; 2 Department of Environmental Biotechnology, Faculty of Biotechnology and Food Sciences, Lodz University of Technology, Wólczańska 171/173, 90-530 Lodz, Poland; 3 Department of Medicine, Surgery and Pharmacy, University of Sassari, Viale San Pietro, I-07100 Sassari, Italy; 4 Faculty of Chemistry, University of Wrocław, F. Joliot-Curie 14, 50-383 Wroclaw, Poland; 5 Department of Occupational Safety Engineering, Faculty of Process and Environmental Engineering, Lodz University of Technology, Wólczańska 213, 90-924 Lodz, Poland; 6 Faculty of Chemistry, Institute of General and Ecological Chemistry, Lodz University of Technology, Żeromskiego 116, 90-924 Lodz, Poland

**Keywords:** genotoxic activity, Schiff bases, copper complexes, flavonoids, HeLa, Caco-2, LLC-PK1

## Abstract

The three Schiff base ligands, derivatives of hesperetin, HHSB (*N*-[2,3-dihydro-5,7-dihydroxy-2-(3-hydroxy-4-methoxyphenyl)chromen-4-ylidene]isonicotinohydrazide), HIN (*N*-[2,3-dihydro-5,7-dihydroxy-2-(3-hydroxy-4-methoxyphenyl)chromen-4-ylidene]benzhydrazide) and HTSC (*N*-[2,3-dihydro-5,7-dihydroxy-2-(3-hydroxy-4-methoxyphenyl)chromen-4-ylidene]thiosemicarbazide) and their copper complexes, CuHHSB, CuHIN, and CuHTSC were designed, synthesized and analyzed in terms of their spectral characterization and the genotoxic activity. Their structures were established using several methods: elemental analysis, FT-IR, UV-Vis, EPR, and ESI-MS. Spectral data showed that in the acetate complexes the tested Schiff bases act as neutral tridentate ligand coordinating to the copper ion through two oxygen (or oxygen and sulphur) donor atoms and a nitrogen donor atom. EPR measurements indicate that in solution the complexes keep their structures with the ligands remaining bound to copper(II) in a tridentate fashion with (O^–^, N, O_ket_) or (O^–^, N, S) donor set. The genotoxic activity of the compounds was tested against model tumour (HeLa and Caco-2) and normal (LLC-PK1) cell lines. In HeLa cells the genotoxicity for all tested compounds was noticed, for HHSB and CuHHSB was the highest, for HTSC and CuHTSC–the lowest. Generally, Cu complexes displayed lower genotoxicity to HeLa cells than ligands. In the case of Caco-2 cell line HHSB and HTSC induced the strongest breaks to DNA. On the other side, CuHHSB and CuHTSC induced the highest DNA damage against LLC-PK1.

## 1. Introduction

Cancer is a leading cause of death worldwide, accounting for nearly 10 million deaths in 2020 [1]. According to World Health Organization (WHO), colorectal cancer is the third most common cancer type worldwide; in 2020, almost 2 million cases were diagnosed. Colorectal cancer is the second most common cause of cancer death, leading to almost 1 million deaths per year. In 2020, an estimated 604,000 women were diagnosed with cervical cancer worldwide and about 342,000 women died from the disease. It is the fourth most common cancer in the whole world [2]. Despite advances in therapy, the treatment of cancer remains unsatisfactory due to resistance developed by the cancer cells to conventional chemotherapeutic drugs. Therefore, the search for new alternatives is necessary.

Schiff bases and their copper(II) complexes have been studied extensively [3,4,5] due to their vital roles in the coordination chemistry inherent from their simple method of preparation and structural variety [6]. Moreover, there are a group of compounds with a wide spectrum of applications in various industries such as food and dye industry, analytical chemistry, catalysis, and agrochemicals [7]. Synthesis of Schiff bases occurs simply and quickly by condensation of a primary amine with a carbonyl group and for this reason as well as for the ability to complex with metal ions, Schiff bases are treated as “privileged ligands” [8,9]. The biological and medicinal activities of Schiff base metal complexes are greater than those of free Schiff bases [10,11].

Hesperetin (5,7,3′-trihydroxyl-4′-methoxyl-flavanone) is a representative of flavonoid which occurs ubiquitously in plants, fruits (especially in citrus fruits), flowers, and foods of plant origin [12]. Hesperetin has multiple biological and pharmacological activities, including antioxidant properties [13] inhibition of cancer development [14,15], effects on the blood–brain barrier [16,17], and signal transduction pathways [18,19]. There are several in vitro studies concerning the anti-proliferative and proapoptotic mechanisms of hesperetin in HeLa (human cervix carcinoma) cell lines, a type of immortalized cervical carcinoma cell line [20,21,22,23,24]. Hesperetin belongs to flavonone and its B ring is not conjugated with the carbonyl group on the ring C, so it gives the possibility of connection of various substituents to the C=O group and formation of new compounds with different biological and pharmacological functions. Furthermore, it is confirmed that the coordination of copper(II) ion with bioactive ligands can actually improve their biological activity, for example Cu(II) complexes with hesperetin, naringenin and apigenin have shown higher inhibitory rate than their free ligands against human SGC-7901 (gastric cancer) and HepG2 (hepatocellular carcinoma) cell lines [21]. Therefore, studying the effect of substituents on hesperetin and its complexity is imperative and can obtain a lot of information on drug actions.

In order to increase the biological activity of hesperetin the conjugation of the flavanone with the hydrazides: benzhydrazide, isoniazid and thiosemicarbazide was carried out. All ligands, HHSB, HIN, and HTSC were evaluated for their electronic and physicochemical properties using experimental and theoretical methods and radical scavenging ability. Based on these studies, it was found that modification of the structure of hesperetin increased its antioxidant activity. While the theoretical DFT(B3LYP) calculations pointed out that in polar solvents (methanol and DMSO) the first step of the free radical scavenging mechanism of hesperetin derivatives was deprotonation of the 7-OH groups leading to the formation of anionic forms. These anionic species were considered to be the entering species to the two coexisting or competing mechanisms: SPLET and SPL-HAT [25]. Among the three ligands listed, only one of them, HHSB, was coordinated with copper(II) acetate, giving CuHHSB complex, and demonstrated in reference [26]. The CuHHSB complex has shown high efficiency in the cleavage of plasmid DNA in an aqueous solution, indicating its potential as a chemical nuclease. Moreover, the CuHHSB complex has greater cytotoxic activity against HeLa and K562 (human erythroleukemia) cells than the HHSB ligand. Additionally, HHSB and CuHHSB display antimicrobial activities against tested strains of bacteria, especially Staphylococcus aureus is more sensitive to CuHHSB [26].

**Figure 1:**
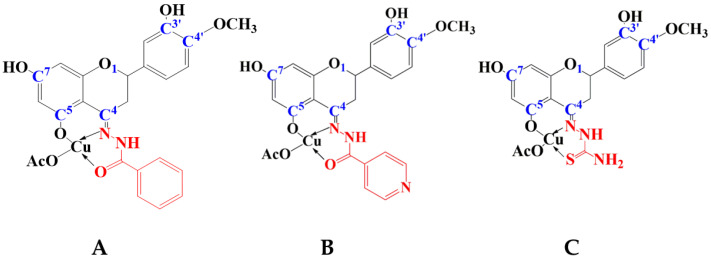
Structure of (**A**) (*E*)-acetoxy((4-(2-benzoylhydrazono)-7-hydroxy-2-(3-hydroxy-4-methoxyphenyl)chroman-5-yl)oxy)copper; (**B**) (*E*)-acetoxy((7-hydroxy-2-(3-hydroxy-4-methoxyphenyl)-4-(2-isonicotinoylhydrazono)chroman-5-yl)oxy)copper (CuHIN); (**C**) (*E*)-acetoxy((4-(2-carbamothioylhydrazono)-7-hydroxy-2-(3-hydroxy-4-methoxyphenyl)chroman-5-yl)oxy)copper (CuHTSC).

In the present work, we have focused on the research of three known ligands HHSB, HTSC and HIN [25], and their copper(II) complexes (CuHHSB, CuHTSC, and CuHIN) (Figure 1). Their structure and physicochemical properties were characterized by spectroscopic (FT-IR, UV-Vis, EPR, ESI-MS) and electrochemical techniques. These compounds were screened for genotoxic activity (basal endogenous DNA damage) against two human tumour cell lines: HeLa (cervix carcinoma) and Caco-2 (colon adenocarcinoma), as well as normal kidney epithelial cells from the pig LLC-PK1. The ability of these compounds to induce oxidative DNA damage, as well as to induce double-strand breaks on HeLa cells was then estimated.

## 2. Results and Discussion

### 2.1. Characterization of the Complexes

#### 2.1.1. IR Spectral Studies

IR spectra provided substantial and valuable information on the coordination reaction. All the spectra recorded were characterized by vibrational bands mainly due to the NH, O–H, C=O, C=N, C=S, and COO^−^ groups (Figure S1) [27,28,29].

The IR spectrum of the Schiff base ligand HHSB (LH_3_) showed a small band at 3613 cm^−1^ due to phenolic OH and medium intensity weak bands at 3173 cm^−1^ due to—amide NH. The strong bands of high intensity observed at 1644, 1601 cm^−1^, and 1264 cm^−1^ were due to the carbonyl function ν(C=O), azomethine function ν(C=N), and phenolic function ν(C–O), respectively. The IR spectra of the metal complex exhibited ligand bands with the appropriate shifts due to a complex formation. It was observed in the [CuLH_3_·OAc]·H_2_O spectrum an absence of stretching vibration due to phenolic OH at 3613 cm^−1^. This may indicate the formation of a coordination bond between the metal ion and phenolic oxygen atom at C5 (Figure S1) via deprotonation. This was further confirmed by the increase in absorption frequency of phenolic ν(C–O) of about 40 cm^−1^ which appeared in the region 1304 cm^−1^ of the complex, indicating the participation of an oxygen atom of the phenolic OH in the coordination. The band assigned to the OH group did not completely disappear, and it was probably merged with the NH band.

The similar situation is the case of the Schiff base ligand HIN (LH_3_). IR spectrum of HIN showed a small band at 3481 cm^−1^ due to phenolic OH and medium intensity weak bands at 3087 cm^−1^ due to—amide NH. The strong bands of high intensity observed at 1659, 1636, and 1246 cm^−1^ were due to the carbonyl function ν(C=O), azomethine function ν(C=N), and phenolic function ν(C–O), respectively. The IR spectra of the metal complex exhibited ligand bands with the appropriate shifts due to a complex formation. It was observed in the [CuLH_3_·OAc]·H_2_O spectrum an absence of stretching vibration due to phenolic OH at 3481 cm^−1^. This may indicate the formation of a coordination bond between the metal ion and phenolic oxygen atom at C5 (Figure S1) via deprotonation. This was further confirmed by the increase in absorption frequency of phenolic ν(C–O) of about 28 cm^−1^ which appeared in the region 1274 cm^−1^ of the complex, indicating the participation of an oxygen atom of the phenolic OH in the coordination. The band assigned to the OH group did not completely disappear, and it was probably merged with the NH band.

IR spectrum of the Schiff base ligand HTSC (LH_3_) presented a small band at 3440 cm^−1^ due to phenolic OH and medium intensity weak bands at 3331 cm^−1^ due to—amide NH. The strong bands of high intensity observed at 1643, 1271/841 and 1163 cm^−1^ were due to the carbonyl function ν(C=N), azomethine function ν(C=S), and phenolic function ν (C–O), respectively. The IR spectra of the metal complex exhibited ligand bands with the appropriate shifts due to a complex formation. During complexation the bands corresponding to ν(O-H), ν(C=N), and ν(–C=S) shifted towards lower side (around ca. 8–44 cm^−1^). This suggests that the ligand acts as bidentate chelating agent coordinating through nitrogen of ν(–C=N) group and sulphur of ν(–C=S) group [30]. In contrast to the bands corresponding to ν(O-H), ν(C=N), and ν(–C=S), the band ν(C-O) shifted towards higher side (around ca. 31 cm^−1^). The increase in absorption frequency of phenolic ν(C–O) which appeared in the region 1194 cm^−1^ of the complex, indicating the participation of an oxygen atom of the phenolic OH in the coordination.

The results clearly indicate the coordination of a copper ion through the N atom of the azomethine group of the HHSB, HTSC, and HIN Schiff base [31]. The presence of acetate anions in the complex was confirmed by the bands of stretching vibrations ν(COO^−^) at the frequencies of 1568 and 1357 cm^−1^ for CuHHSB; 1547 and 1357 cm^−1^ for CuHTSC; and 1538 and 1375 cm^−1^ for CuHIN. Coordination of the metal ion with the ligand was additionally confirmed by the appearance in the spectrum of the complex of new, non-ligand bands of weak intensity in the regions of 443 and 418 cm^−1^ for CuHHSB, 457 and 377 cm^−1^ for CuHTSC, and 508 and 481 cm^−1^ for CuHIN assigned to the stretching vibrations ν(M–O) and ν(M–N), where M is a metal ion, respectively.

#### 2.1.2. UV-Vis Studies

In the UV absorption spectra of flavonoid, including hesperetin, there are generally two main absorption bands, band I (300–400 nm) and band II (240–300 nm) [32], which are associated with the absorption of the cinnamoyl system and the absorption of the benzoyl moiety in the molecules, respectively. Figure S2 shows the UV absorption spectra of ligands and complexes systems in DMSO solvent. The absorption bands, appearing in hesperetin derivatives spectra, can be assigned on the basis of the literature data on absorption spectra of Schiff bases [33]. The band I at λ_max_ = 324 in HHSB, at λ_max_ = 329 in HIN and HTSC could be assigned to the π–π* transition within the C=N group. The bands II in the range of high energy, at λ_max_ = 260 nm in HHSB, λ_max_ = 259 and 288 nm in HIN and λ_max_ = 261 and 281 nm in HTSC, are due to the excitation of the π electrons (π–π* transitions) of the aromatic rings. [26].

In the spectra of the metal complexes, λ_max_ for all bands was shifted to higher wavelengths. In the range of band I for CuHHSB, λ_max_ was shifted to a higher wavelength by approximately 60 nm compared to that of the ligand, for CuHIN–67 nm and for CuHTSC–11 and 39 nm. In band II, slight shifts of λ_max_ were recorded, namely 1 nm for CuHHSB, 8 nm for CuHIN, and 12 nm for CuHTSC. The observed shifts in both bands confirmed that the reaction had occurred between Cu^2+^ and the ligands [34].

#### 2.1.3. PXRD Studies

Powder X-ray diffraction (PXRD) experiments were carried out on the hesperetin ligands and their Cu-complexes at room temperature. (Figure S3). All samples of Cu complexes turned out to be crystalline (CuHHSB and CuHTSC) or partially crystalline (CuHIN). The X-ray diffractometry patterns of the obtained complexes have completely different patterns in comparison to ligands, thus confirming the existence of new compounds.

#### 2.1.4. TGA Studies

To determine the thermal stability of ligands and their complexes, the thermogravimetric (TGA) analysis was performed (Figure S4). Each tested compound exhibited weight loss in two steps; the first one appeared in the temperature range of 24–249 °C with a weight loss of 4.2–11.2 % for ligands; for complexes 30–308 °C with a higher weight loss of 11.0–31.4 % (Table S1). This may be because of the evaporation of the free water and ligand and complex water molecules. The second weight loss occurred above 250 °C for HTSC, 221 and 248 °C for HHSB and HIN, respectively, due to the decomposition of the organic ligand and led to a weight loss as it was presented in Table S1. In case of all complexes the highest temperature of decomposition was observed. In the sample of CuHIN the beginning of decomposition process was noticed at 282.9 °C, CuHHSB–292.8 °C and for CuHTSC 309.5 °C. Their weight losses during the decomposition step are shown in the Table S1.

#### 2.1.5. EPR Studies

Many recent papers highlighted that the major species present at low metal concentration could be completely different from the complex initially introduced in the incubation medium and, for this reason, the determination of the active species and mode of action is not straightforward [35].

**Figure 2:**
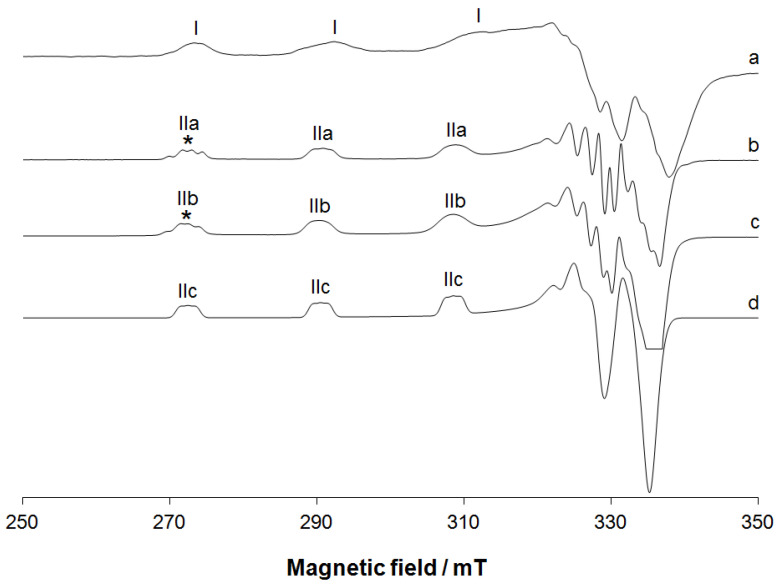
X-band anisotropic EPR spectra recorded at 120 K on the polycrystalline complex [Cu(HHSB)(AcO)]: (**a**) solid powder; (**b**) dissolved in DMF; (**c**) dissolved in DMSO and (**d**) dissolved in a mixture H_2_O/DMSO 60/40 *v*/*v*. With **I**, **IIa**, **IIb** and **IIc** the resonances of [Cu(HHSB)(AcO)], [Cu(HHSB)(DMF)]^+^, [Cu(HHSB)(DMSO)]^+^ and [Cu(HHSB)(H_2_O)]^+^ are indicated. With an asterisk the superhyperfine coupling with ^14^N nucleus is also denoted.

**Scheme 1:**
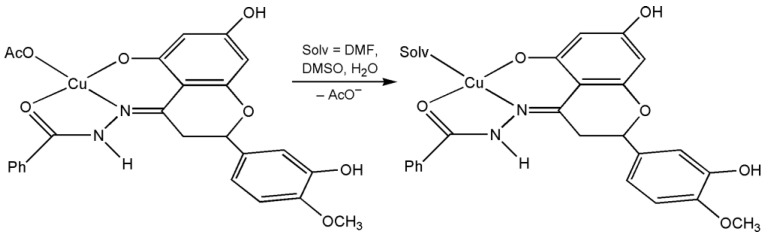
Reaction of [Cu(HHSB)(AcO)] in DMF, DMSO or H_2_O.

EPR spectrum of the polycrystalline solid complex [Cu(HHSB)(AcO)] was recorded at 120 K and is shown in trace **a** of Figure 2. In contrast with what was observed for other spectra in the solid state [36,37,38,39], the hyperfine coupling between the unpaired electron and ^63,65^Cu nuclei (I = 3/2) is revealed. The EPR spectrum can be attributed to only one species with g_z_ = 2.230 and A_z_ = 198.6 × 10^−4^ cm^−1^ and a coordination mode (O^−^, N, O_ket_); AcO^−^ (**I** in Figure 2). When the solid sample is dissolved in DMF or DMSO, an increase in g_z_ and a decrease in A_z_ is observed (g_z_ = 2.243−2.244 and A_z_ = 189.6–190.4 × 10^−4^ cm^−1^). This suggests that the monodentate AcO^–^ ligand is replaced by the less basic solvent molecules (Scheme 1). The species formed, [Cu(HHSB)(DMF)]^+^ and [Cu(HHSB)(DMSO)]^+^, are indicated by **IIa** and **IIb** in traces **b** and **c** of Figure 2. Overall, the spin Hamiltonian parameters determined are similar to those measured in a mixture H_2_O/DMSO 60/40 *v/v* (trace **d** in Figure 2 and Table 1), where the coordination of water ligand occurs [26].

**Figure 3:**
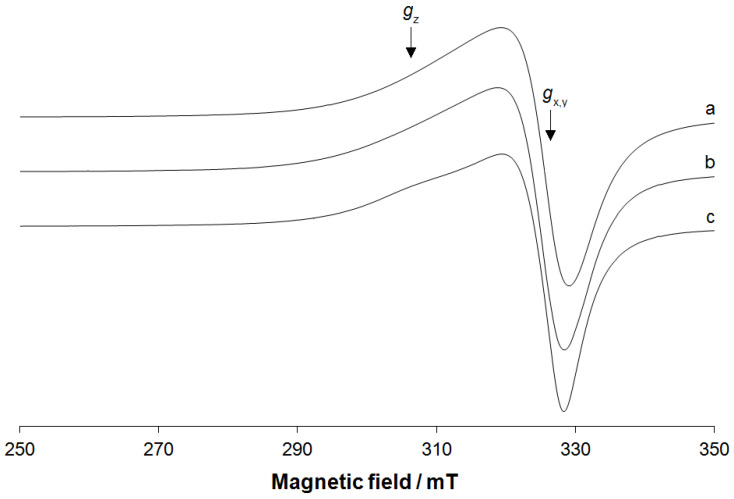
X-band anisotropic EPR spectra recorded at 120 K on the polycrystalline complex {[Cu(HIN)](AcO)}_n_: (**a**) solid powder; (**b**) dissolved in DMF and (**c**) dissolved in DMSO. With the arrows the position of *g*_z_ and *g*_x_, *g*_y_ is indicated.

**Figure 4:**
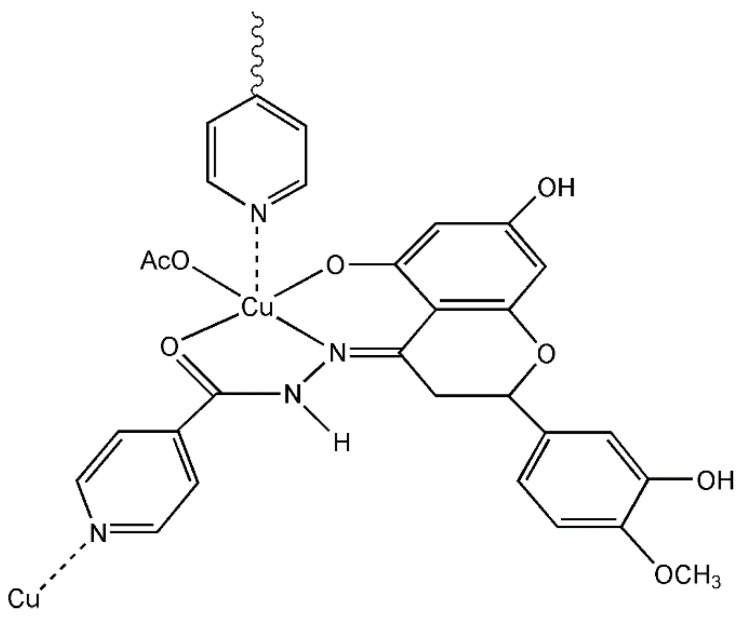
Structure of the polynuclear species {[Cu(HIN)](AcO)}_n_.

EPR spectrum recorded at 120 K on the polycrystalline powder of {[Cu(HIN)](AcO)} were characterized by two g values in the order g_z_ > g_x_ ~ g_y_ > g_e_ (trace **a** of Figure 3). These values are consistent with a d_x2–y2_ ground state and a square planar geometry [41,42]. As pointed out recently in the literature, the broad signal is due to a mixture of intercenter exchange and dipolar interactions [43]. Interestingly, when the solid compound is dissolved in on organic solvent such as DMF or DMSO, the spectra do not change and the hyperfine coupling with copper nuclei, typical of solution spectra, is not resolved. The values of g_z_ and g_y_~g_x_ remain almost unchanged and in DMSO solution they can be measured with accuracy (Table 2). This indicates that the structure in the solid state is retained and that the copper ions continue to interact magnetically. These findings can be explained postulating the formation of a polymeric structure with the pyridine-N binding Cu(II) ion of another unit and bridging two different copper centres in the axial position, the equatorial sites being occupied by three HIN donors and acetate ion, as demonstrated by IR spectroscopy (Figure 4). As pointed out recently in the literature, this is possible for Cu(II) complexes [44]. Therefore, the correct formulation of the complex formed by HIN may be {[Cu(HIN)](AcO)}_n_ to indicate its polynuclear nature.

The characteristic signal at half-field (centred at g = 4.28, Figure S5), due to the forbidden ∆M_s_ = *±*2 transition [45], confirms the magnetic interaction between neighbouring copper(II) ions and the existence of a polynuclear species in the organic solution.

**Figure 5:**
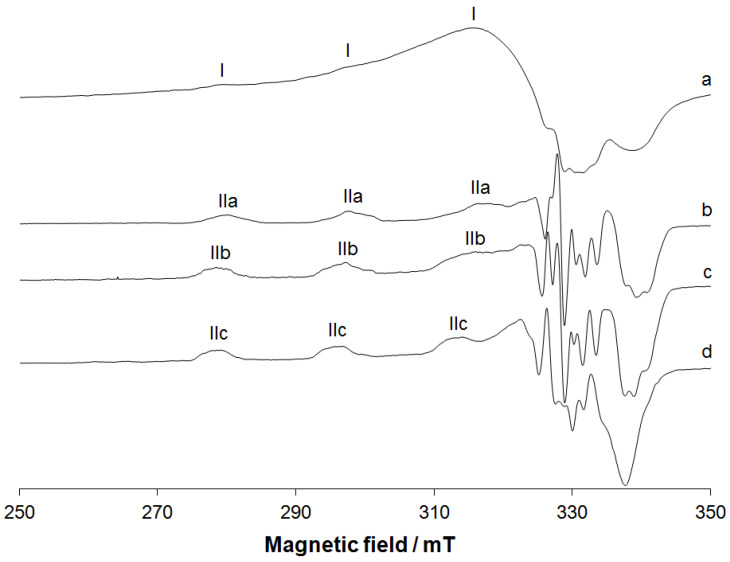
X-band anisotropic EPR spectra recorded at 120 K on the polycrystalline complex [Cu(HTSC)(AcO)]: (**a**) solid powder; (**b**) dissolved in DMF and (**c**) dissolved in DMSO. In (**d**) the spectrum recorded on a solution containing CuSO_4_ and HTSC with ratio 1/1 at pH 7.90 is reported for comparison. With **I**, **IIa**, **IIb** and **IIc** the parallel resonances of [Cu(HTSC)(AcO)], [Cu(HTSC)(DMF)]^+^, [Cu(HTSC)(DMSO)]^+^ and [Cu(HTSC)(H_2_O)]^+^ are indicated.

The solid compound [Cu(HTSC)(AcO)] was recently characterized in the literature [46]. The geometry around copper is square planar with HTSC which behaves as a tridentate ligand with (O^−^, N, S) coordination mode and an acetate anion which completes the equatorial plane. However, in contrast with was observed in our previous study, the EPR spectrum of the solid complex is broad and the hyperfine coupling of the unpaired electron with ^63,65^Cu nuclei is detected (**I** in trace a of Figure 5). The spin Hamiltonian parameters are g_z_ ~ 2.20 and A_z_ ~ 185 × 10^−4^ cm^−1^, very similar to those measured previously (g_z_ = 2.200 and A_z_ = 185.0 × 10^−4^ cm^−1^) [46].

In an organic solution (DMF, DMSO) the parameters change slightly (Table 1) and the variation can be due to the fact that a solvent molecule replaces AcO^−^ ion. If in these systems the acetate ion would remain bound to the copper, the same values of g_z_ and A_z_ would have expected. Instead, the spin Hamiltonian parameters are comparable but not coincident with those of the similar species [Cu(HTSC)(H_2_O)]^+^ [40], formed in an aqueous solution containing CuSO_4_ and HTSC in a molar ratio 1/1 (i.e., in a system without AcO^−^). The first three parallel resonances of [Cu(HTSC)(H_2_O)]^+^ are indicated with **IIc** in trace **d** of Figure 5, while those of the species [Cu(HTSC)(DMF)]^+^ and [Cu(HTSC)(DMSO)]^+^ are denoted by **IIa** and **IIb** in traces **b** and **c**. It can be noticed that the first three parallel of resonances are broad, but the superhyperfine coupling with ^14^N nucleus cannot be resolved, differently from the systems with HHSB (cfr. Figure 2 and Figure 5). In this case, the value of the linewidth of the low-field parallel hyperfine resonance (∆H in Table 1) allows us to distinguish how many nitrogens are bound to Cu^II^ ion: the values in the range 3.7–4.1 mT for [Cu(HTSC)(DMF)]^+^, [Cu(HTSC)(DMSO)]^+^ and [Cu(HTSC)(H_2_O)]^+^ suggests the coordination of only one nitrogen donor. For comparison, for the coordination of two nitrogens a value significantly larger, around 5.0–6.0 mT, is expected [47].

#### 2.1.6. Biological Activity

Detection of damage to deoxyribonucleic acid (DNA) at the cellular level is important in the field of genotoxicity testing, environmental/human bio-monitoring, diagnosis of genetic disorders, etc. Single cell gel electrophoresis (SCGE) or the comet assay is a versatile and sensitive technique used to measure DNA damage and repair in individual cells. Comet assay is one of the methods with high cognitive potential. The comet assay helps to measure the single/double-strand DNA breaks, alkali labile sites (apurinic/apyrimidinic sites), base-pair damages, and apoptotic nuclei in the cells. A wide range of samples, including peripheral blood, cultured cells, cancer cells, yeast cells, and bacteria, can be subjected to SCGE. The most widely used method for assessment of DNA damage is the alkaline comet assay; hence in the present work, it was used to assess DNA breakage in HeLa cells that were exposed to various concentrations of HHSB, HTSC, HIN, CuHHSB, CuHIN, and CuHTSC. Treatment of agarose-embedded cells with hypertonic lysis solution and non-ionic detergent removes their cell membranes, cytoplasm, nucleoplasm, and dissolves nucleosomes. Subsequently, when the leftover nucleoid is treated with a high alkaline solution, DNA supercoils unwind/relax thereby exposing the alkali labile sites (apurinic/apyrimidinic sites) which appear as breaks. Such breaks migrate towards the anode when exposed to the current during electrophoresis thereby producing a ‘comet’-like appearance. The level of this kind of DNA damage was assessed by the use of endonuclease III (Endo III). Endo III converts oxidized pyrimidines into strand breaks, which can be detected by the comet assay [48].

- Basal endogenous DNA damage

As expressed by DNA percentage in the comet tail, the non-exposed cells in DMEM (negative control) exhibited DNA damage of 5.4% ± 0.6% (HeLa and LLC-PK1) and 3.2% ± 0.4% (Caco-2). The treatment of the cells with 50 µM hydrogen peroxide (positive control) resulted in 50.3% ± 0.6% (HeLa), 42.0% ± 3.6% (Caco-2), and 54.1% ± 0.2% (LLC-PK1) damage.

The genotoxic activity of the HHSB, HIN and HTSC ligands, as well as their Cu complexes was tested against model tumour (HeLa and Caco-2) and normal (LLC-PK1) cell lines in three concentrations: 1, 10, and 50 µM. Cells incubated with different concentration of cisplatin served as a reference sample.

**Figure 6:**
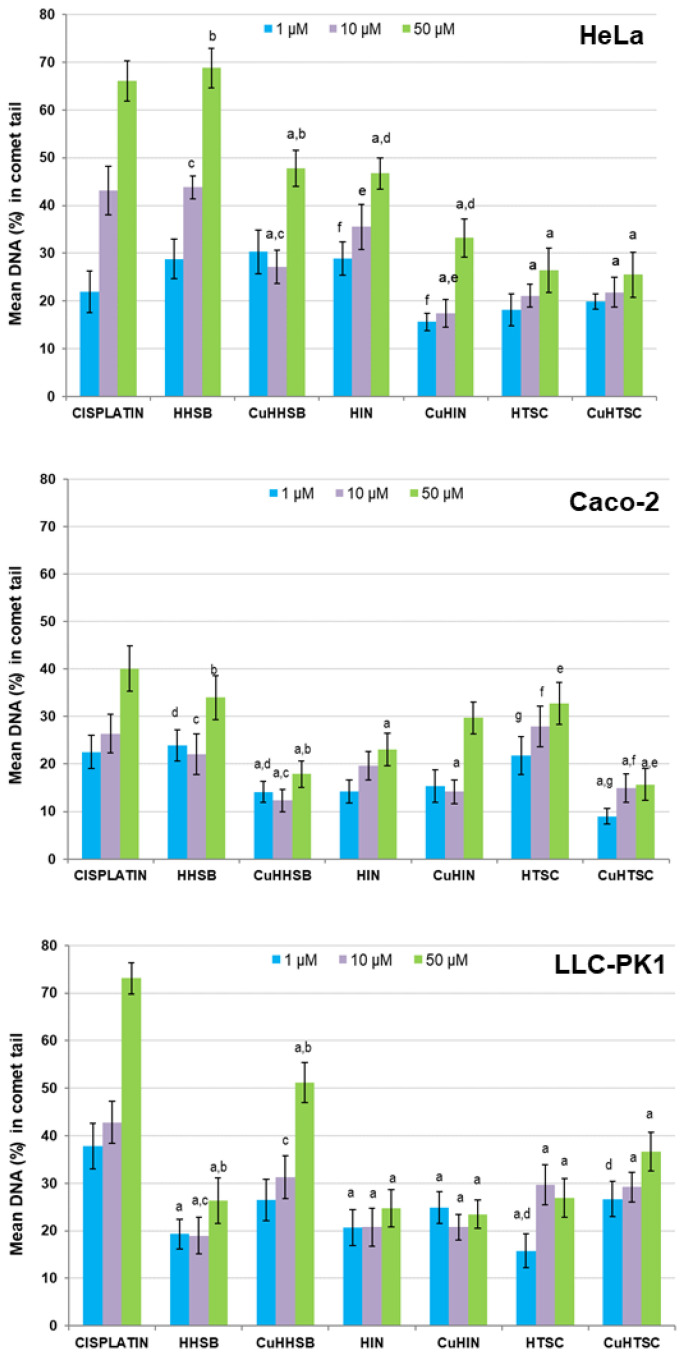
Basic endogenous DNA damage in different cell lines after exposition to tested compounds, expressed as percentage of DNA in the tail of comets in the alkaline comet assay. The number of cells analysed in each treatment was 50. Error bars denote standard error of the mean (S.E.M). Results significantly different from: ^a^ cisplatin, ^b–g^ Cu ligand; ANOVA (*p* ≤ 0.05).

In HeLa cells the genotoxicity of cisplatin and HHSB was the highest and comparable and correlated with tested concentrations (Figure 6). It ranged from 21.9% ± 4.4% to 66.1% ± 4.2% and from 28.8% ± 4.1% to 68.8% ± 4.1% for cisplatin and HHSB, respectively. Both HHSB and CuHHSB complex showed genotoxic potency, but HHSB showed greater genotoxic activity, than CuHHSB what was better demonstrated in case of higher concentrations–10 and 50 µM (*p* ≤ 0.05). In case of 1 µM the differences were not noticeable, 28.8% ± 4.1% and 30.3% ± 4.6% for HHSB and CuHHSB, respectively. Moreover, CuHIN displayed lower genotoxicity to HeLa cells, than HIN. DNA damage induced by 1 µM of CuHIN was almost two times lower (15.6% ± 1.8%) than in the presence of HIN (28.9% ± 3.5%) (*p* ≤ 0.05). The genotoxicity of HTSC and CuHTSC was the slightest of all tested compounds and remained at the similar level. Generally, concentration dependent rise in the extent of DNA damage was observed in the presence of tested chemicals.

In the case of the Caco-2 cell line (Figure 6), 50 µM cisplatin induced the strongest breaks to DNA (40.1% ± 4.8%), while genotoxicity of lower doses was comparable to that of HHSB and HTSC. Moreover, both–HHSB and HTSC in all doses, demonstrated higher genotoxic activity than their Cu ligands (*p* ≤ 0.05), and in the case of 50 µM dosage it was two-times higher: it accounted for 34.0% ± 4.6% for HHSB and 32.8% ± 4.4% for HTSC, and 17.9% ± 2.8% and 15.7% ± 3.4% for their ligands, respectively (*p* ≤ 0.05). The highest dose of CuHIN (50 µM) induced greater DNA damage (29.7% ± 3.3%) than HIN (23.1% ± 3.4%), which was not observed for 1 and 10 µM.

For LLC-PK1 (Figure 6), cisplatin was the most genotoxic in all tested doses, and for 50 µM it reached 73.1% ± 3.3% (*p* ≤ 0.05). Cu complexes of HHSB and HTSC induced greater DNA damage than HHSB and HTSC. In the case of 50 µM CuHHSB the DNA damage was doubled (from 26.3% ± 4.8% to 51.2% ± 4.2%) (*p* ≤ 0.05). Genotoxic potential of HIN and CuHIN was at the similar level regardless of the dosage.

Generally, Caco-2 cells seem to exhibit lower susceptibility to the tested compounds than HeLa and LLC-PK1 cells. As HeLa cells demonstrated the highest sensibility to tested substances, they were taken for further analysis of DNA break types (DSBs and oxidative).

**Figure 7:**
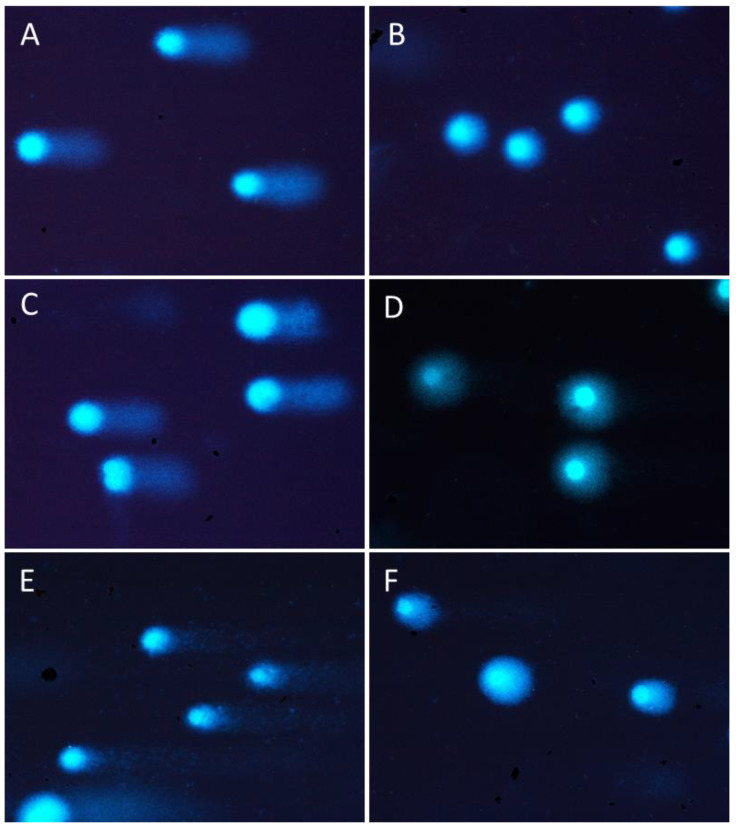
Representative photographs of DNA damage of DAPI (4′,6-diamidino-2-phenylindole) stained comets in HeLa cells: (**A**)—50 µM cisplatin, (**B**—negative control, (**C**)—50 µM HHSB; in Caco-2 cells: (**D**)—10 µM CuHHSB; in LLC-PK1 cells: (**E**)—50 µM CuHHSB, and (**F**)—50 µM HHSB. Fluorescence microscopy (Nikon, Tokyo, Japan), objective 20×).

Representative comets were presented in Figure 7.

- DSBs induction

The genotoxic activity was tested in two repeats. The non-exposed HeLa cells in DMEM (negative control) exhibited no DNA damage (3.4% ± 0.3%). As cisplatin directly interacts with multiple cellular components including proteins, peptides, and nucleic acids it also forms DNA adducts by covalent binding to primarily purine bases of DNA. It results in mono-, inter-, and intra-strand adducts, causing distortion of the DNA double helix, which can block DNA replication and transcription [49]. Cisplatin is a typical control for DSBs. In the research it induced DSBs yielding 30.2% ± 3.4% (Table 3). HHSB, HIN, and HTSC induced higher DNA DSBs than their Cu ligands, among which HHSB inducted the highest amount of DSBs–23.3% ± 1.0%. The results between Cu ligands and appropriated non-ligands were statistically significant (*p* ≤ 0.05), except for HIN and CuHIN. All results were also statistically significant in comparison to cisplatin (*p* ≤ 0.05), except for HHSB. The results of neutral comet assay suggest that tested chemicals might induce DSBs in HeLa cells. DSBs can lead to DNA mutations such as chromosome aberrations [50].

- Endogenous oxidative DNA damage

Endonuclease III was employed as part of the research. It converts oxidized pyrimidines into strand breaks, which can be detected by the comet assay. The results indicate solely the DNA base modification, which are not alkali-labile. The extent of oxidative DNA damage in HeLa cells recognised by Endo III was the highest in the case of the positive control–50 µM hydrogen peroxide (data not presented)–it was 26.2% ± 4.6%. For the reference sample–50 µM cisplatin it was at the similar level–26.5% ± 3.9% (Table 3). The non-exposed HeLa cells in DMEM (negative control) exhibited no DNA damage (5.6% ± 2.0%). All tested compounds exhibited weak oxidative DNA damage towards HeLa cells, with the highest genotoxicity in the case of CuHIN (12.2% ± 4.2%). The results were significant (*p* ≤ 0.05) in comparison to cisplatin, but there were no significant differences among HHSB, HIN, HTSC, and their Cu ligands.

**Figure 8:**
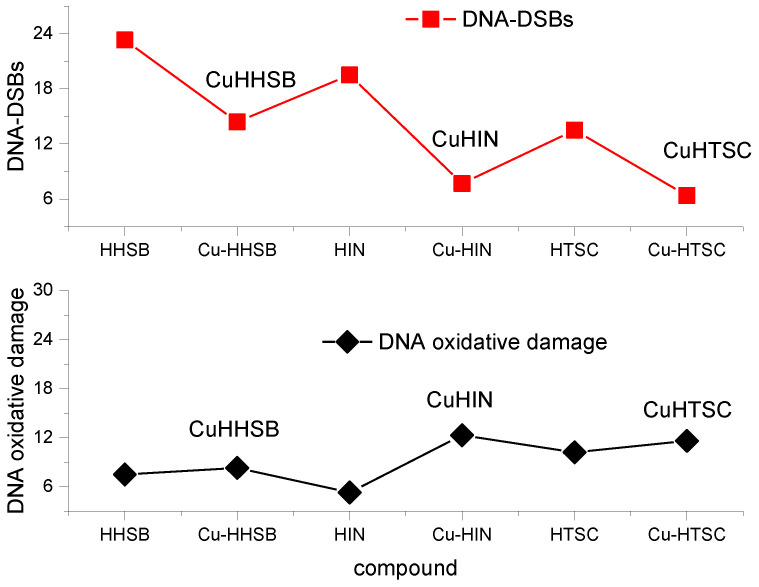
DNA oxidative effect of ligands and copper complexes.

The presented results demonstrate the pro-oxidant activity of the tested compounds. The highest pro-oxidant activity was shown by HTSC among the ligands and CuHIN between the complexes. Generally, copper complexes exhibit greater oxidative damage activity than ligands. Ligands and copper complexes induce DSBs through, at least in part, oxidative mechanisms, and these compounds can interfere with DSBs repair (Figure 8).

Metal–organic frameworks (MOFs) are being studied for their antibacterial, anticancer, and antiinflammation activity [51]. Strong antimicrobial activity was verified for silver-MOFs both for Gram-positive (e.g., *Staphylococcus aureus*, *Bacillus subtilis*) and Gram-negative (e.g., *Pseudomonas aeruginosa*, *Escherichia coli*) bacteria, as well as for *Candida albicans* yeast [52]. MOFs play important role in the anticancer therapy. For example, it was shown, that Ti-tetrakis(4-carboxyphenyl)porphyrin (TCPP) the induced generation of high amounts of reactive oxygen species (ROS) in pancreatic carcinoma cell line BxPC-3. Additionally, it induced direct DNA damage in vitro (double strand breaks as in our study) leading to apoptosis and induced cell cycle arrest in S phase [51]. Several mechanisms of anticancer activity of MOFs were demonstrated widely for manganese based MOFs, which combine with cancer cells DNA and proteins to induce apoptosis, bind to DNA through intercalation, cause DNA lysis, promote apoptosis of cancer cells, induce cell cycle arrest in GO/G1 phase, inhibit cancer cells migration, and increase ROS levels [53,54,55,56]. ROS (e.g., especially hydroxyl and superoxide radicals) penetrate cell membrane and then can react with macromolecules such as DNA and as a result, can induce cancer cell death (apoptosis or necrosis) [57]. In current research CuHIN, HTSC, and CuHTSC induced weak oxidative DNA damage, which indicates a weak induction of oxidative stress. Khabour et al. [58] studied the genotoxicity (sister chromatid exchanges and chromosomal aberrations) of two Schiff complexes to either Cu(II) or Zn(II) in the human lymphocyte cultures. They proved that the tested complexes were genotoxic to varying degrees, depending on the compound, the concentration, and the assay. In the highest concentration tested (10 µg/mL) Cu(II) complexes induced slightly higher chromosomal aberrations and sister chromatid exchanges, than Zn(II). In another research, pyrimidine Schiff base genotoxicity was evaluated on gastric adenocarcinoma cells (AGS) [59]. The complexes (50 µM) induced significant single- and double-strand DNA breaks that was measured with the comet assay. Comparing the results obtained in our current study with the works of other authors involves some difficulty, due to the dissimilarity of the compounds/complexes, different concentrations tested and the cell lines used. Nevertheless, Schiff bases and their complexes with metals show biological activity by inducing DNA damage in cancer cells of various types.

Numerous studies regarding metal complexes of Schiff bases and their biological properties are being performed around the world. Rao and co-workers [60] have synthesized and characterized a benzothiazole based Schiff base and their metal complexes (Cu(II), Co(II), Ni(II), Zn(II), and Cd(II)). Their antibacterial and cytotoxic tests showed that all metal complexes showed better activity than the corresponding ligand. Moreover, among all the complexes, Cu complex exhibited potent activity compared to other complexes. Another example, the copper–Schiff base complex of carvacrol, a major component of most essential oil-bearing plants, confirms that complex was exceptionally active against A549 cells as compared to carvacrol [61]. Bansal and co-workers stated that the presence of the copper ion (Cu^2+^) significantly enhanced the cytotoxic activity by targeting the delivery of the ligand to the cancer cells. In the case of flavonoids and their derivatives, and more specifically Schiff bases, complexation reactions with various metals are carried out. Namely, complexes of copper (II) with hesperetin, naringenin, and apigenin of general composition [CuL_2_(H_2_O)_2_] · nH_2_O were tested in vitro against human cancer cell lines hepatocellular carcinoma (HepG-2), gastric carcinomas (SGC-7901), and cervical carcinoma (HeLa). Their results showed that complexes hesperetin and apigenin exhibited higher inhibitory rate than their free ligands against SGC-7901 and HepG2 cell lines [21]. For a novel Schiff-base ligand (H_5_L), hesperetin-2-hydroxy benzoyl hydrazone, and its copper (II), zinc (II), and nickel (II) complexes (M·H_3_L) [M(II) = Cu, Zn, Ni] performed studies (DNA binding) confirmed that complex systems are more effective than their ligands. Metal complexes of hesperetin derivatives possess better antioxidant activity against superoxide and hydroxyl radicals than the free ligand [62]. Another example that relates to hesperetin Schiff bases is a hesperetin Schiff base ligand (H_4_L) and its complexes, [H_3_CuL·OAc]·H_2_O and [H_3_ZnL·OAc]·2H_2_O [34]. Li and Yang also pointed out the better DNA binding and the antioxidant activity (O2^-•^ and HO^•^) is noticed for copper complexes of novel hesperetin Schiff bases than their ligands.

## 3. Materials and Methods

### 3.1. Materials in the Synthesis of Copper Complexes with Hesperetin Derivatives

The racemic hesperetin, benzhydrazide, 2-aminobenzhydrazide, isoniazid, thiosemicarbazide, and copper(II) acetate monohydrate, and all other compounds were purchased from Sigma-Aldrich Co. (Poznań, Poland). All reagents were of analytical quality and were used without further purification.

### 3.2. Apparatus

The structure of the compounds obtained was determined by: elemental analysis (C, H, N) on the EuroVector 3018 analyzer (EuroVector, Milan, Italy); and analysis of the IR spectra using an FT-IR spectrometer Nicolet 6700 (Thermo Scientific, Waltham, MA, USA) in the range of 4000–350 cm^−1^. UV-Vis spectra of the compounds in the DMSO and the mixture of 70% HCl(NaCl)/30% were recorded in the λ interval 200–900 nm using a Hewlett-Packard 8453 spectrophotometer running 845x UV-Visible ChemStation Software (Agilent, Mulgrave, Victoria, Australia). Solutions were inserted in a quartz cell with a path length of 1 cm. The content of copper in the complex was determined by a 932 AA spectrophotometer from GBC (Dandenong, VIC, Australia) with a deuterium background correction used. The system was controlled by a data station running the GBC Avanta Version 1.33 (GBC, 932 AA spectrophotometer, Dandenong, VIC, Australia). The formation of the new compounds was monitored by electrospray ionization mass spectrometry (ESI-MS, Bruker Daltonics GmbH, Bremen, Germany) analysis in the negative ion mode. Before the analysis, the samples were dissolved in acetonitrile (250 µg/mL) and injected directly into a Q-Exactive Orbitrap™ (Thermo Scientific, Bellefonte, PA, USA) tandem mass spectrometer equipped with a heated electrospray ionization (ESI) interface (HESI–II), using an injection pump and a 500 µL syringe (Thermo Scientific, Bellefonte, PA, USA). The injection speed was 10 µL/min. The new compounds were analysed using full-scan MS and a subsequent parallel reaction monitoring (PRM, Thermo Scientific, Hudson, NH, USA) mode with a scan range from 50 to 1000 *m*/*z*. The capillary temperature was adjusted to 320 °C. The electrospray capillary voltage and S-lens radio frequency (RF) level were set at 4.5 kV and 50 V, respectively. Nitrogen was used as both a sheath gas and auxiliary gas at a flow of 10 and 8 (arbitrary units), respectively. Ions that were selected by the quadrupole entered the higher energy collision dissociation (HCD) cell. An isolation window of 2 amu was used and the precursors were fragmented by a collision-induced dissociation C-trap (CID) with a normalized collision energy (NCE) of 25 V. The ESI-MS/MS scan spectra (Bruker Daltonics GmbH, Bremen, Germany) were acquired with the mass resolution of 35,000 full-width at half-maximum (FWHM) at m/z = 100. The automatic gain control (AGC) target (the number of ions to fill C-Trap) was set at 2.0 × 105 with a maximum injection time (IT) of 100 ms. The instrument control, data acquisition, and evaluation were completed with the Q Exactive Tune 2.1 and Thermo Xcalibur 2.2 software (Thermo Fisher Scientific, Bremen, Germany). Room temperature powder X-ray diffraction (PXRD) patterns were collected using a PANalytical X’Pert Pro MPD diffractometer (Malvern Panalytical Ltd., Royston, UK) in Bragg-Brentano reflecting geometry with (CuKα) radiation from a sealed tube. Data were collected in the 2θ range of 5–90° with a 0.0167° step and 30 s exposure per step. Thermogravimetric analyses (TGA) were measured on a TG 209 F3 Tarsus thermogravimetric analyser (Netzsch, Selb, Germany) with a heating rate of 10 K/min (from 25 to 900 °C) under an N_2_ atmosphere with the flow rate 20 mL/min.

### 3.3. Characterization of Ligands

The synthesis and characterization of HHSB were presented in reference [26]. Whereas HIN and HTSC syntheses and their description were described in reference [25].

All ligands (HHSB, HIN and HTSC) have been prepared according to the literature procedure [34].

### 3.4. Characterization of Copper(II) Complexes in Solid State

#### 3.4.1. Characterization of Copper(II) Complex with HHSB in Solid State

The synthesis and characterization of CuHHSB was presented in reference [26].

#### 3.4.2. Characterization of Copper(II) Complex with HIN in Solid State

An amount of 60.0 mg (0.14 mmol) of hesperetin acylhydrazone derived from isoniazid (HIN) was quantitatively transferred to a round bottom flask and dissolved in 20 mL of DMSO/ethanol (5:20) system at 45 °C. The contents of the flask were stirred for an hour until the HIN was completely dissolved, then 28.65 mg (0.14 mmol) of Cu(OAc)_2_·H_2_O was added to the obtained solution, with three drops of triethylamine added as a catalyst, and the reaction was carried out at 60 °C within four hours. After this time, a dark brown copper complex precipitated, which was suction filtered, washed with acetone and distilled water, and left to dry in the desiccator for 48 h in the presence of drier (calcium chloride). The synthesis yield was 63%.

For the [CuLH_2_(OAc)] (CuHIN as abbreviation); Yield: 47.63 mg, 63%; Anal. Calc. C_25_H_23_CuN_3_O_7_: C, 55.50; H, 4.29; N, 7.77; Cu, 11.75. Found: C, 53.81; H, 4.14; N, 7.51; Cu, 11.33%. IR *ν*_max_ (cm^−1^): *ν*(N–H): 3233, *ν*(C=O): 1609, *ν*(C=N): 1582, *ν*_as_(COO^−^): 1538, *ν*(C=C):1439, *ν*_s_(COO^−^): 1375, *ν*(C–O): 1274, *ν*(C–O–C): 1148, *ν*(C–N): 1072, *ν*(N–N): 1021, *ν*(M–O): 508, *ν*(M–N): 481. UV–Vis λ_max_ (nm): 261, 332, 398 for 70% HCl(NaCl)/30% DMSO and 267, 396 for 100% DMSO (Figure S2). ESI–MS: *m*/*z* = 543.

#### 3.4.3. Characterization of Copper(II) Complex with HTSC in Solid State

An amount of 38.15 mg (0.10 mmol) of hesperetin thiosemicarbazone (HTSC) was quantitatively transferred to a round bottom flask and dissolved in 15 mL of ethanol at 45°C. The contents of the flask were stirred for 10 min until the HTSC was completely dissolved, then 22.80 mg (0.11 mmol) of Cu(OAc)_2_·H_2_O was added to the obtained solution and the reaction was carried out at 45 °C within three hours. After this time, a brown copper complex precipitated, which was suction filtered, washed with acetone and distilled water, and left to dry in the desiccator for 48 h in the presence of drier (calcium chloride). The synthesis yield was 84.9%.

For the [CuLH_2_(OAc)] (CuHTSC as abbreviation); Yield: 67.83 mg, 84.9%; Anal. Calc. C_19_H_19_CuN_3_O_7_S: C, 45.92; H, 3.85; N, 8.46; S, 6.45; Cu, 12.79. Found: C, 45.71; H, 3.87; N, 8.45; S, 6.39; Cu, 12.48%. IR *ν*_max_ (cm^−1^): *ν*(O–H): 3432, *ν*(N–H): 3233, *ν*(C=N): 1600, *ν*_as_(COO^−^): 1547, *ν*(C=C): 1433, *ν*_s_(COO^−^): 1357, *ν*(C=S): 1253/814, *ν*(C–O): 1194, *ν*(C–O–C): 1128, *ν*(C–N): 1065, *ν*(N–N): 1021, *ν*(M–S): 457, *ν*(M–N): 377. UV–Vis λ_max_ (nm): 244, 333 for 70% HCl(NaCl)/30% DMSO and 273, 340, 368 for 100% DMSO (Figure S2). ESI–MS: *m*/*z* = 437 [M–H]^+^ (M without acetate and water molecule). (Mass spectrum is available as Figure S6).

### 3.5. EPR Experiments

EPR spectra were recorded on the polycrystalline powder or on the solid complexes dissolved in aqueous or organic solution in the temperature range 120–298 K with an X-band Bruker EMX spectrometer equipped with an HP 53150A microwave frequency counter. The microwave frequency was 9.40–9.41 GHz at 120 K and 9.83–9.85 GHz at 298 K; microwave power was 20 mW; time constant was 81.92 ms; modulation frequency 100 kHz, modulation amplitude 0.4 mT; the resolution was 4096 points.

### 3.6. Genotoxic Activity

#### 3.6.1. Cell Culture

Human cervix (HeLa, ATCC, USA) and human colon adenocarcinoma (Caco-2, Cell Lines Service, Eppelheim, Germany) were used as a model tumour adherent cell lines in the study. Additionally, pig kidney epithelial cell line LLC-PK1 (Cell Lines Service, Eppelheim, Germany) was applied as a model of normal cells. The HeLa cells were a gift from a professor who wished to remain anonymous. Caco-2 cells were from passage 43, while LLC-PK1 cells were from passage 38.

All cells were cultured in Roux flasks (Becton, Dickinson and Co., Franklin Lakes, NJ, USA) as a monolayer. HeLa and Caco-2 cells were cultured in Dulbecco’s Modified Eagle’s Medium (DMEM, Sigma-Aldrich, St. Louis, MO, USA) complemented with 10% foetal bovine serum (FBS, Gibco, Thermo Fisher Scientific, Waltham, MA, USA), 4 mM GlutaMAX^TM^ (Gibco, Thermo Fisher Scientific, Waltham, MA, USA), 25 mM HEPES (Sigma-Aldrich, St. Louis, MO, USA), 100 μg/mL streptomycin and 100 IU/mL penicillin (Sigma-Aldrich, St. Louis, MO, USA). LLC-PK1 were cultured in Dulbecco’s Modified Eagle’s Medium/Ham’s F12 (DMEM/Ham’s F12, 1:1; Cell Lines Service, Eppelheim, Germany) with 5% FBS (Cell Lines Service, Eppelheim, Germany), supplemented with 2 mM L-glutamine (Cell Lines Service, Eppelheim, Germany) and HEPES (Cell Lines Service, Eppelheim, Germany). All cells were cultured for 7–10 days at 37 °C in a 5% CO_2_ atmosphere and humidity >95% in a CO_2_ incubator (Galaxy 48S, New Brunswick, United Kingdom). The cells were washed every 2–3 days with PBS (pH 7.2, Sigma-Aldrich, St. Louis, MO, USA) and a fresh medium was added. After reaching the confluence cells were detached with TrypLE^TM^ Express (Gibco, Thermo Fisher Scientific, Waltham, MA, USA) for 5–10 min at 37 °C according to manufacturer’s instruction, suspended in sterile PBS and aspirated off the plastic flask. As the enzyme is of plant origin the reaction does not demand to be terminated by the addition of FBS. The cell suspension was centrifuged (187× *g*, 5 min). The pellet was re-suspended in a fresh culture medium. Subsequently, the cell count was performed with the use of haemocytometer, and the viability of cells was determined by trypan blue (Sigma-Aldrich, St. Louis, MO, USA) exclusion. The cells were ready to use if the viability was min. 90%.

#### 3.6.2. Single-Cell Gel Electrophoresis Assay (Comet Assay)

The final concentration of HeLa/Caco-2/LLC-PK1 cells in each sample was adjusted to 10^5^ cells/mL. 900 µL of cells in non-supplemented DMEM were incubated with 100 µL of each compound at 37 °C for 1 h. All stock concentrations of tested compounds were freshly prepared in non-supplemented DMEM just before the addition to cells. The final tested concentrations of the compounds were: 1, 10, and 50 µM. Tested concentrations were chosen based on previous cytotoxicity studies [26]. The concentrations were ≤IC_50_ and close to IC_0_, so they were moderate, weakly, or no cytotoxic for cells.

The comet assay was performed under alkaline conditions (pH > 13) according to the procedure of Singh et al. [63] with some modifications [64,65]. After incubation, the cells were centrifuged (182× *g*, 15 min, 4 °C), decanted, suspended in 0.75% LMP (low melting point) agarose (Sigma-Aldrich, St. Louis, MO, USA), layered onto slides precoated with 0.5% NMP (normal melting point) agarose (Sigma-Aldrich, St. Louis, MO, USA), and lysed at 4 °C for 1 h in a buffer consisting of 2.5 M NaCl, 1% Triton X-100, 100 mM EDTA, and 10 mM Tris, pH 10. After the lysis, the slides were placed in an electrophoresis unit and DNA was allowed to unwind for 20 min in an electrophoretic solution containing 300 mM NaOH and 1 mM EDTA. Electrophoresis was conducted at 4 °C for 20 min at an electric field strength of 0.73 V/cm (300 mA). Then, the slides were neutralized with distilled water, dried overnight, stained with 1.0 µg/mL DAPI (4′,6-diamidino-2-phenylindole) (Sigma-Aldrich, St. Louis, MO, USA), and covered with cover slips. The slides were examined at 200× magnification under a fluorescence microscope (Nikon, Tokyo, Japan) connected to a video camera and a personal computer-based image analysis system–Lucia-Comet v. 7.0 (Laboratory Imaging, Prague, Czech Republic). Fifty images were randomly selected from each sample and the percentage of DNA in the comet tail was measured.

#### 3.6.3. Double-Strain Breaks

The ability of tested compounds (at concentration of 50 µM) to induce DNA double-strand breaks (DSBs) in model HeLa cells was evaluated by the neutral comet assay according to Błasiak et al. (2012) [66]. In this version of the comet assay electrophoresis ran in a buffer consisting of 100 mM Tris and 300 mM sodium acetate at pH 9.0 (with glacial acetic acid). Electrophoresis was conducted for 60 min, after a 20 min equilibrium period, at an electric field strength of 0.41 V/cm (50 mA) at 4 °C. The slides were then proceeded as described in the “Single-cell gel electrophoresis assay” section.

#### 3.6.4. Oxidative DNA Damage

Oxidative DNA damage was evaluated with the DNA repair enzyme–endonuclease III (Endo III, New England Biolabs Inc., Germany) in model HeLa cells. The procedure was performed according to [67]. To estimate the ability of the enzyme to recognize oxidized DNA bases, the cells were incubated either with tested compounds (at concentration of 50 µM), or hydrogen peroxide (50 µM), lysed and post-treated with Endo III. After lysis slides were washed three times in an enzyme buffer (composed of 40 mM HEPES-KOH, 0.1 KCl, 0.5 mM EDTA, 0.2 mg/mL bovine serum albumin, pH 8.0) and drained, and the agarose was covered with 25 µL of either enzyme buffer or enzyme at 1 µg/mL in buffer, sealed with a cover glass and incubated for 30 min at 37°C. Further steps were described above. To determine the net value of DNA damage recognized by the enzyme, the DNA damage observed in the absence of the enzyme was subtracted from that measured in the presence of it.

#### 3.6.5. Statistical Analysis

Comet data were analysed using two-way analysis of variance (ANOVA), while a particular mode of interaction × time was used to compare the effects induced by chemicals at this mode of interaction. The differences between samples with the normal distribution were evaluated the Student’s test. Student’s test and ANOVA was conducted using OriginPro 6.1 software to evaluate the experimental data. Significant differences were accepted at *p* ≤ 0.05 as calculated with Duncan’s multiple range test (Statistica 10, StatSoft). The results are presented as mean ± standard error of the mean (S.E.M).

## 4. Conclusions

Flavanones are considered as one of the most important and well-known heterocyclic nuclei, which is a common and integral feature of a variety of natural products and medicinal agents. The work described in this paper involved the synthesis, physicochemical characterization as well as biological activities of novel Schiff bases derived by condensation of hesperetin with benzhydrazide, isoniazide and thiosemicarbazide. Their chelating ability towards copper ions were also investigated.

IR spectra provided the coordination reaction between hesperetin Schiff bases and copper(II) acetate. In the acetate complexes, the tested Schiff bases act as neutral tridentate ligand coordinating to the copper ion through two oxygen, in the case of HHSB and HIN, (or oxygen and sulphur in the case of HTSC) donor atoms and nitrogen donor atom.

For Cu(II) complexes formed by HHSB, HIN, and HTSC, the EPR spectra indicate that in solution the ligands bind copper in a tridentate fashion with (O^−^, N, O_ket_) or (O^−^, N, S) donor set and that the ligand should be retained in biological media. Only the acetate ion equatorially coordinated to copper can be replaced by a solvent molecule such as H_2_O, DMF and DMSO. Therefore, the biological activity detected for these complexes may be attributed to the administered complexes and, in particular, to the metal moiety [CuL]^+^ with L = HHSB, HIN, and HTSC.

Our results indicate prooxidant activity of the compounds studied. Most pronounced oxidative activity revealed HTSC between the ligands and CuHIN between the complexes. Generally, copper complexes have a greater oxidative damaging effect than the ligands. Ligands and copper complexes induced DSBs through, at least in part, oxidative mechanisms, and these compounds may interfere with DSBs repair.

These results show that the synthesized compounds exhibit cytotoxic effects against cancer cells and that the cause of cell death is due to DNA damage. In conclusion, this study shows that the studied compounds are suitable for anticancer activity screening studies. In addition, the obtained results can be a basis for further research on the biological activity of hesperetin Schiff bases and their complexes with metal ions.

## Figures and Tables

**Table 1:** Spin Hamiltonian parameters of copper(II) complexes formed by HHSB and HTSC.

Species	*g*_z_	*A*_z_/10^−4^ cm^−1^	∆H/mT ^a^	Coordination Mode
[Cu(HHSB)(AcO)]	2.230	198.6	3.6	(O^−^, N, O_ket_); AcO^–^
[Cu(HHSB)(DMF)]^+^	2.243	189.8	3.8	(O^−^, N, O_ket_); O_DMF_
[Cu(HHSB)(DMSO)]^+^	2.244	190.4	3.7	(O^−^, N, O_ket_); O_DMSO_
[Cu(HHSB)(H_2_O)]^+^	2.243	188.8	3.8	(O^−^, N, O_ket_); O_water_ ^b^
[Cu(HTSC)(AcO)]	~2.20	~185	^c^	(O^−^, N, S); AcO^–^
[Cu(HTSC)(DMF)]^+^	2.181	192.7	3.9	(O^−^, N, S); O_DMF_
[Cu(HTSC)(DMSO)]^+^	2.194	190.6	4.1	(O^−^, N, S); O_DMSO_
[Cu(HTSC)(H_2_O)]^+^	2.200	185.0	3.7	(O^−^, N, S); O_water_ ^d^

^a^ Linewidth of the low-field parallel hyperfine resonance. ^b^ Parameters taken from ref. [26]. ^c^ Value not measurable. ^d^ Parameters taken from ref. [40].

**Table 2:** Spin Hamiltonian parameters of copper(II) complex formed by HIN.

Species	System	*g*_z_	*g*_x_, *g*_y_	Coordination Mode
{[Cu(HIN)](AcO)}_n_	Powder	~2.19	2.063	(O^−^, N, O_ket_); AcO^–^; N_pyr_^ax^
{[Cu(HIN)](AcO)}_n_	DMF	~2.20	2.064	(O^−^, N, O_ket_); AcO^–^; N_pyr_^ax^
{[Cu(HIN)](AcO)}_n_	DMSO/DMF	~2.20	2.063	(O^−^, N, O_ket_); AcO^–^; N_pyr_^ax^
{[Cu(HIN)](AcO)}_n_	DMSO	2.192	2.062	(O^−^, N, O_ket_); AcO^–^; N_pyr_^ax^

**Table 3:** DNA damage ± standard error of the mean (S.E.M.) in HeLa cells after exposition to tested compounds, (50 μM) expressed as percentage of DNA in the tail of comets recognised by Endo III enzyme (oxidative DNA damage) and in the neutral (double-strand breaks) comet assay. The number of cells analysed in each treatment was from 50 to 100. Results significantly different from ^a^ cisplatin; ^b,c^ Cu ligand, ANOVA (*p* ≤ 0.05).

Compound	Mean DNA (%) in Comet Tail (±S.E.M.) of HeLa Cells	
	DSBs	Endo III
Cisplatin	30.2 ± 3.4	26.5 ± 3.9
HHSB	23.3 ± 1.0 ^a^	7.5 ± 3.9 ^a^
CuHHSB	14.4 ± 0.7 ^a,b^	8.3 ± 2.7 ^a^
HIN	19.5 ± 0.7 ^a^	5.3 ± 3.5 ^a^
CuHIN	7.7 ± 3.6 ^a^	12.2 ± 4.2 ^a^
HTSC	13.5 ± 0.7 ^a,c^	10.2 ± 2.0 ^a^
CuHTSC	6.4 ± 2.3 ^a,c^	11.6 ± 1.9 ^a^

## Data Availability

Not applicable.

## Supplementary Materials

The supporting information can be downloaded at: https://www.mdpi.com/article/10.3390/ijms24010761/s1.
