# Supplementary material for: Spectroscopic Characterization and Biological Activity of Hesperetin Schiff Bases and Their Cu(II) Complexes
Source: Int J Mol Sci. 2023 Jan 1;24(1):761. doi: 10.3390/ijms24010761 (PMC9821237; doi:10.3390/ijms24010761)
Supplement: Supplementary file 1 [file ijms-24-00761-s001.zip › ijms-2097783-Table S1.pdf]

**Table S1.** Thermal analytical data of ligands and complexes.

| Compound. | Dehydration temperature [°C]. | | | Decomposition temperature [°C]. | | |
|---|---|---|---|---|---|---|
| | T1 | T2 | Weight loss [%] | T3 | T4 | Total weight loss [%] |
| **HHSB** | 35,70 | 220,00 | 11,15 | 221,40 | 347,40 | 68,57 |
| **HIN** | 24,50 | 247,00 | 6,01 | 248,80 | 335,30 | 84,82 |
| **HTSC** | 35,00 | 249,00 | 4,20 | 250,70 | 353,20 | 99,86 |
| **CuHHSB** | 40,00 | 291,00 | 11,03 | 292,80 | 538,90 | 88,50 |
| **CuHIN** | 30,00 | 281,00 | 19,09 | 282,90 | 448,30 | 59,75 |
| **CuHTSC** | 30,00 | 308,00 | 31,43 | 309,50 | 479,60 | 65,99 |

T1-T2: Temperature range corresponding to complex dehydration; T3-T4 temperature range corresponding to complex decomposition.
