# Supplementary figures and images for: Spectroscopic Characterization and Biological Activity of Hesperetin Schiff Bases and Their Cu(II) Complexes
Source: Int J Mol Sci. 2023 Jan 1;24(1):761. doi: 10.3390/ijms24010761 (PMC9821237; doi:10.3390/ijms24010761)

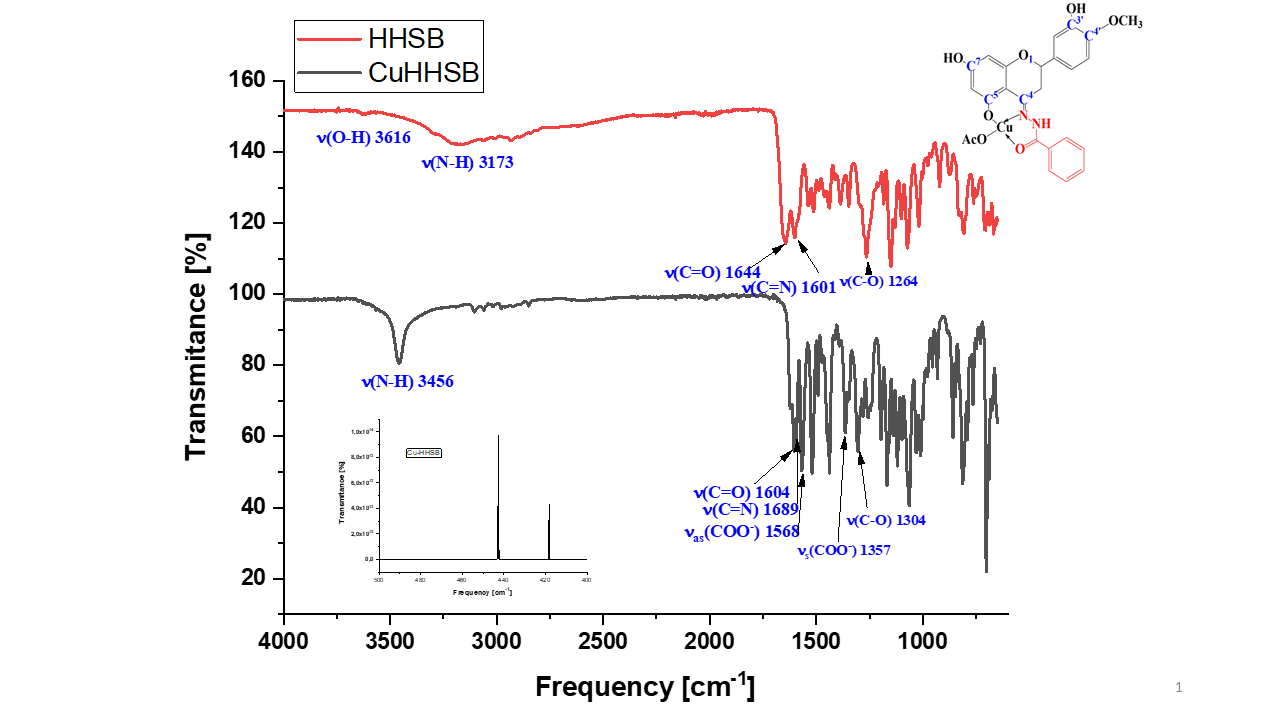

Supplement: Supplementary file 1 [file ijms-24-00761-s001.zip › Figure S1 HHSB and CuHHSB.TIF]

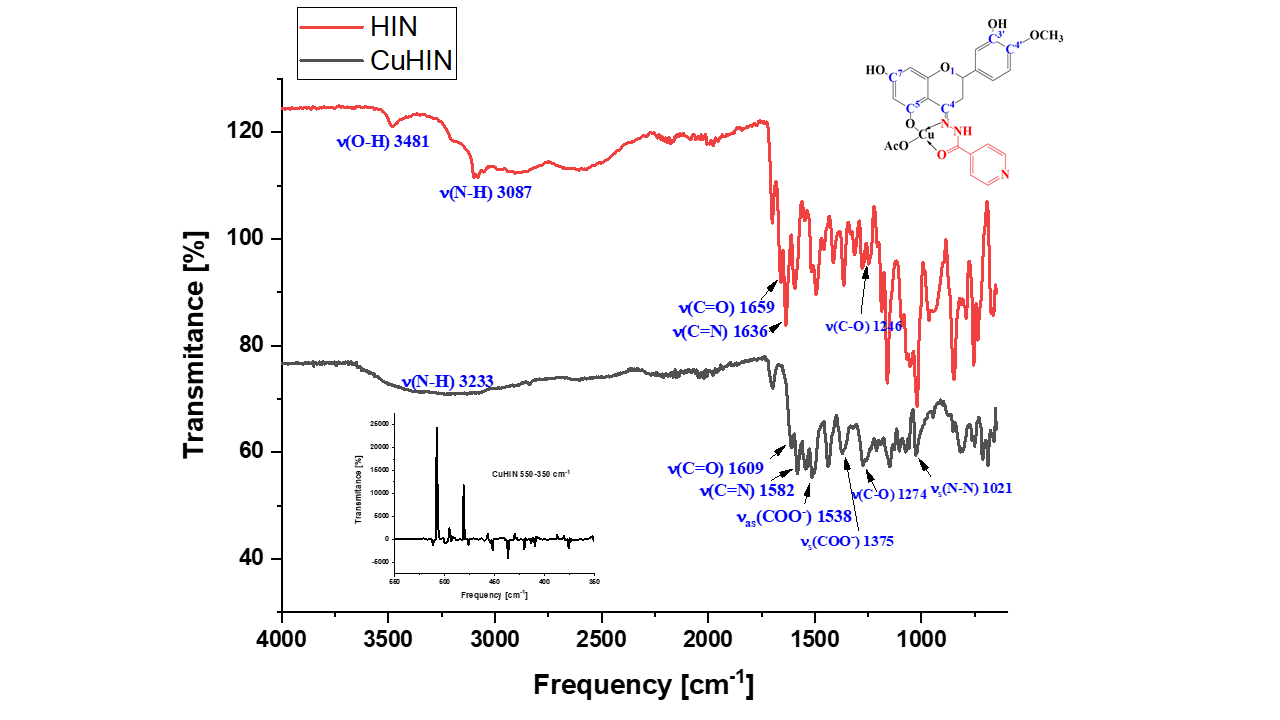

Supplement: Supplementary file 1 [file ijms-24-00761-s001.zip › Figure S1 HIN and CuHTSC.TIF]

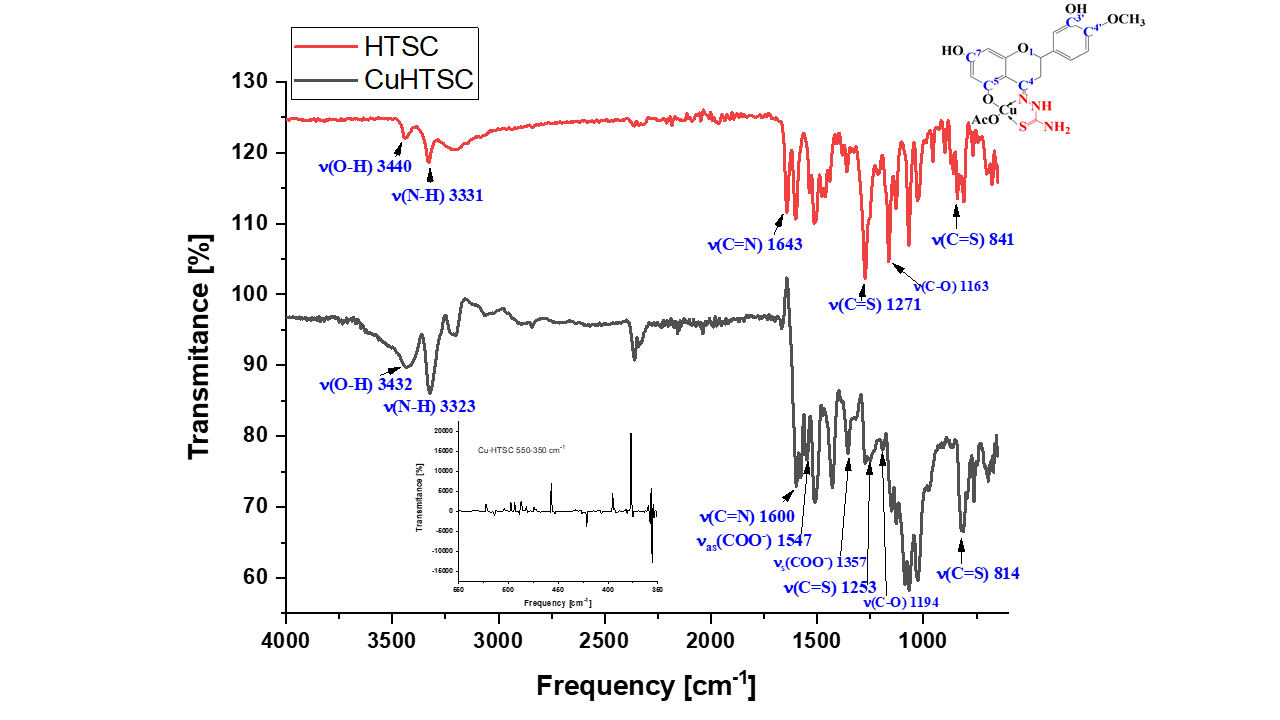

Supplement: Supplementary file 1 [file ijms-24-00761-s001.zip › Figure S1 HTSC and CuHTSC.TIF]

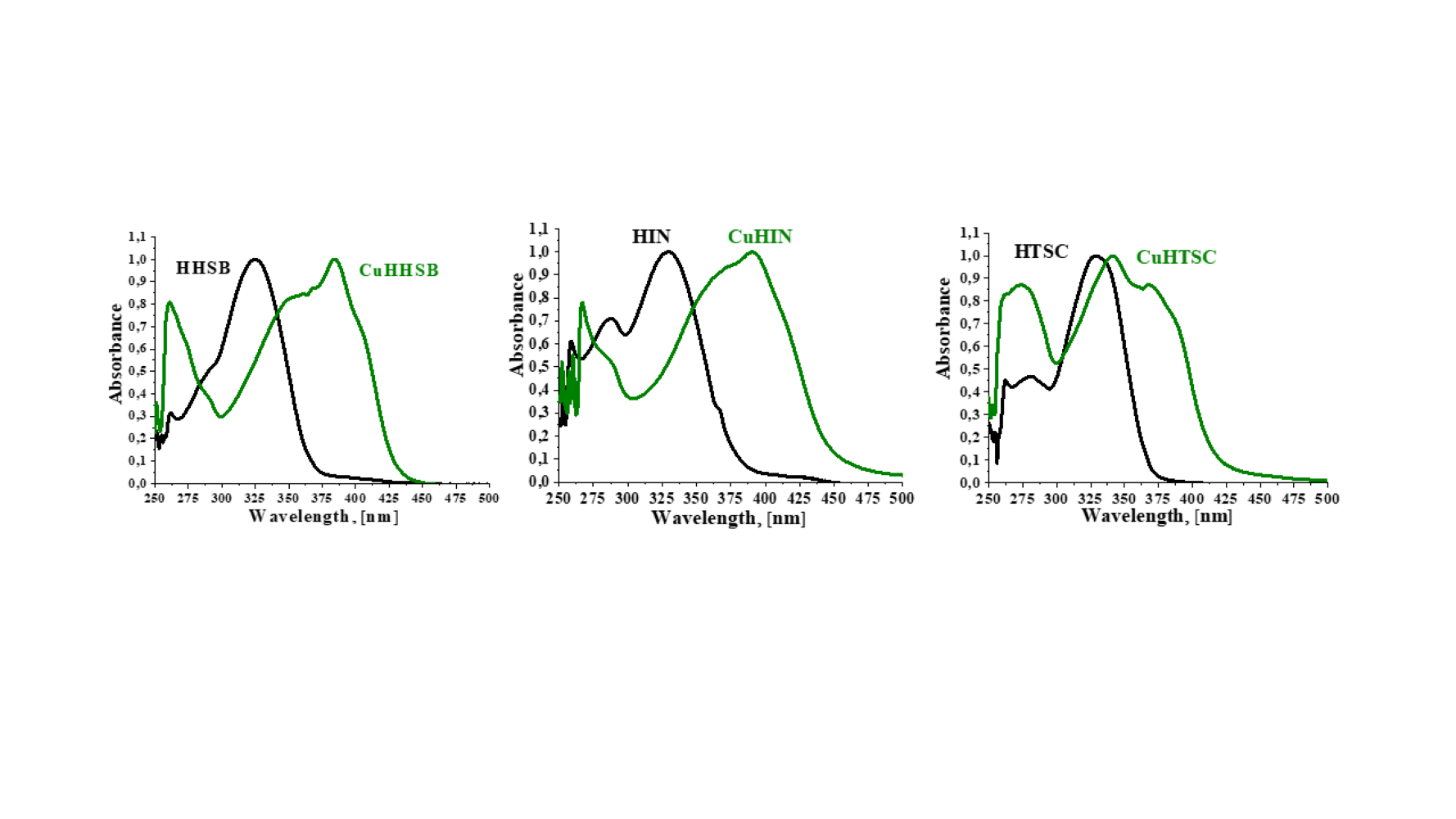

Supplement: Supplementary file 1 [file ijms-24-00761-s001.zip › Figure S2.tif]

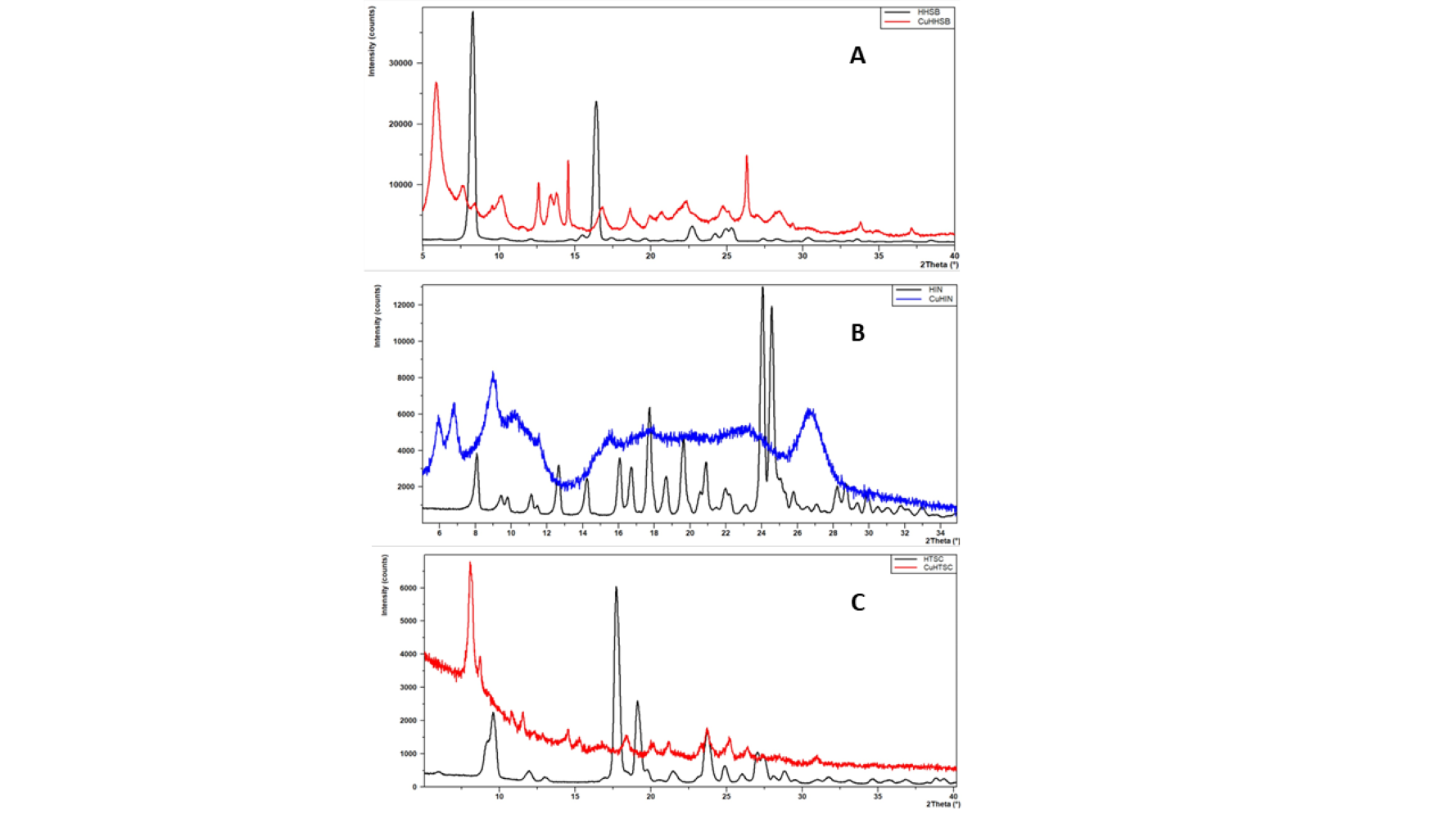

Supplement: Supplementary file 1 [file ijms-24-00761-s001.zip › Figure S3.tif]

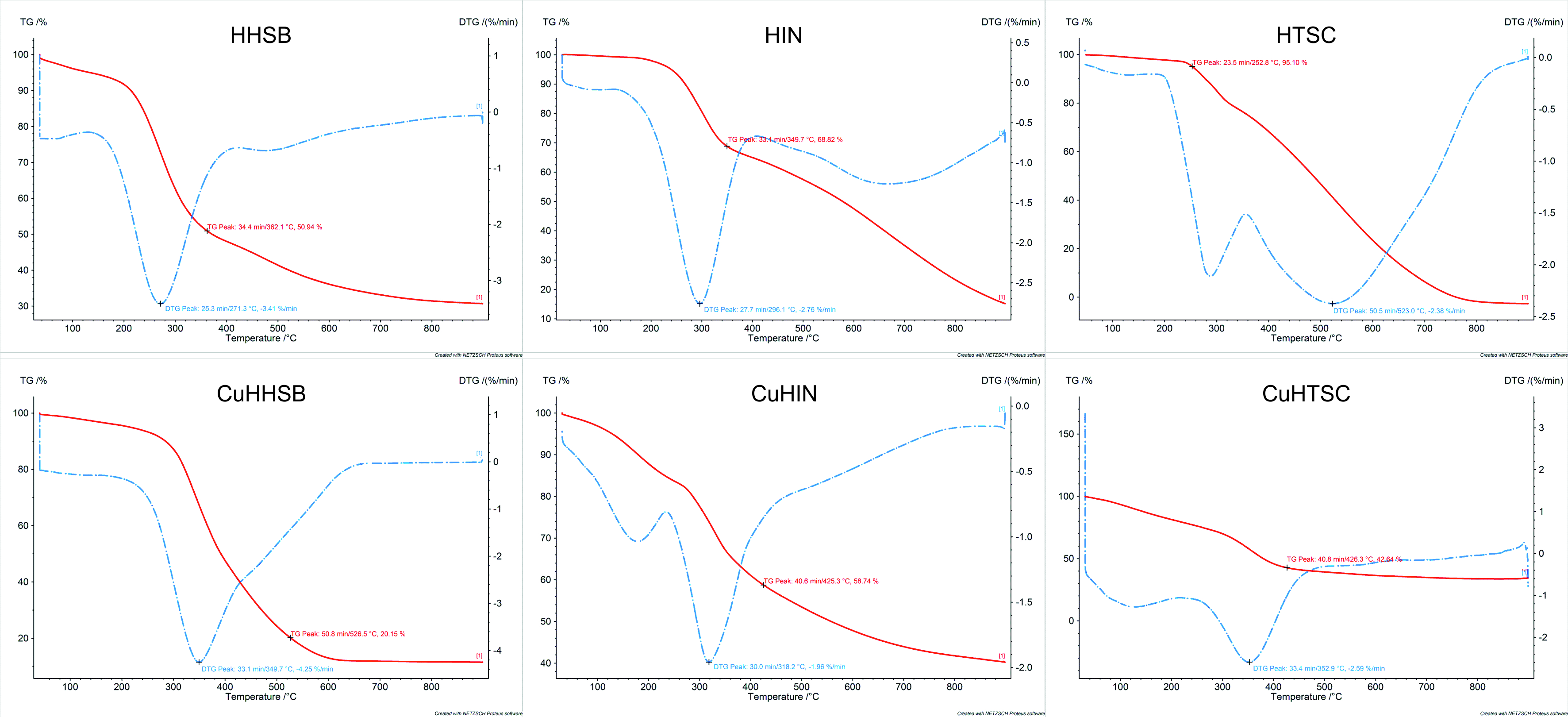

Supplement: Supplementary file 1 [file ijms-24-00761-s001.zip › Figure S4.jpg]

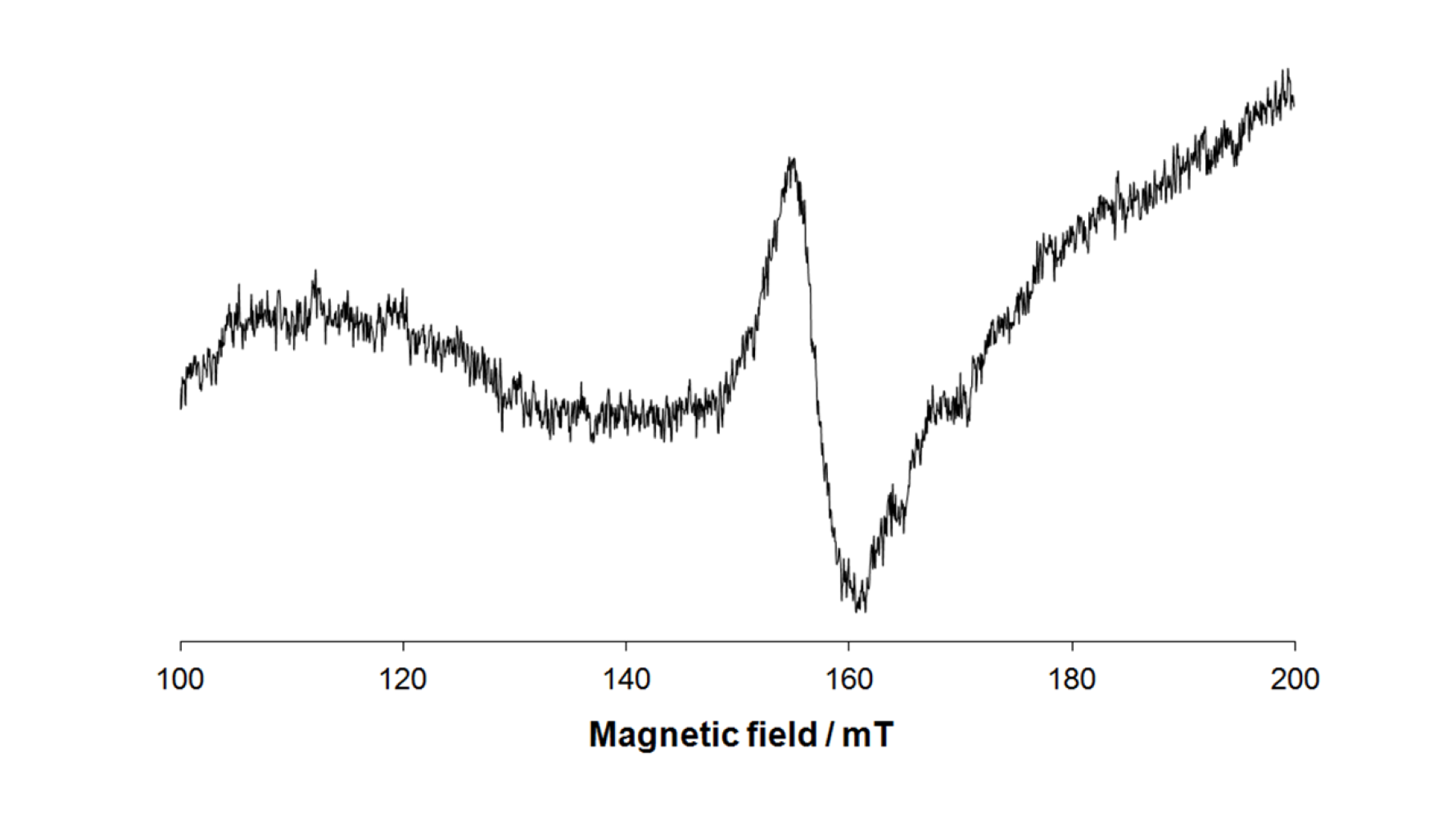

Supplement: Supplementary file 1 [file ijms-24-00761-s001.zip › Figure S5.tif]

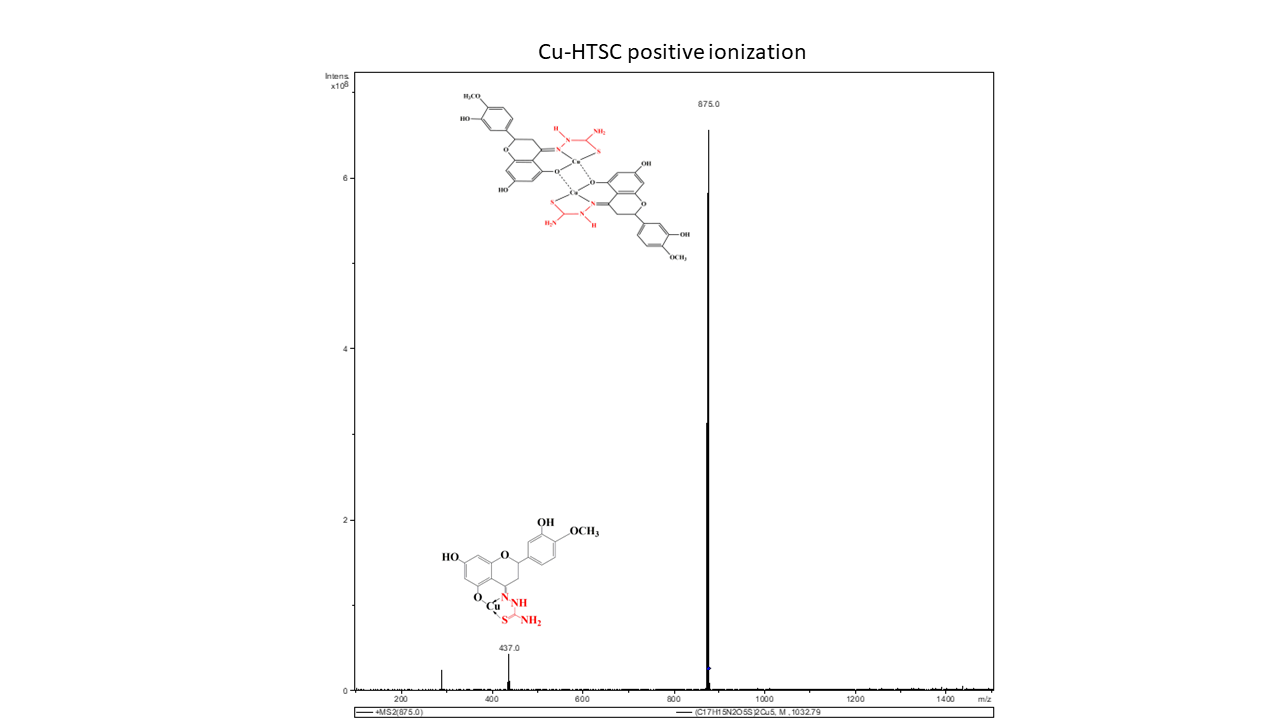

Supplement: Supplementary file 1 [file ijms-24-00761-s001.zip › Figure S6.tif]
